# Which Sudden Stratospheric Warming Events Are Most Predictable?

**DOI:** 10.1029/2022JD037521

**Published:** 2022-09-16

**Authors:** Dvir Chwat, Chaim I. Garfinkel, Wen Chen, Jian Rao

**Affiliations:** ^1^ Fredy and Nadine Herrmann Institute of Earth Sciences Hebrew University Jerusalem Israel; ^2^ College of Earth and Planetary Sciences University of Chinese Academy of Sciences Beijing China; ^3^ Institute of Atmospheric Physics Chinese Academy of Sciences Beijing China; ^4^ Key Laboratory of Meteorological Disaster, Ministry of Education (KLME) Joint International Research Laboratory of Climate and Environment Change (ILCEC) Collaborative Innovation Center on Forecast and Evaluation of Meteorological Disasters (CIC‐FEMD) Nanjing University of Information Science and Technology Nanjing China

**Keywords:** sudden warmings, MJO, ENSO, subseasonal predictability

## Abstract

The predictability of Northern Hemisphere sudden stratospheric warming (SSW) events is considered in 10 subseasonal to seasonal (S2S) forecast models for 16 major SSWs that have occurred since 1998, a larger sample size than has been considered by previous works. The four factors that most succinctly distinguish those SSWs with above average predictability are a preconditioned vortex prior to the SSW, an active Madden‐Julian Oscillation with enhanced convection in the West Pacific, the Quasi‐Biennial Oscillation phase with easterlies in the lower stratosphere, and the vortex morphology (displacement more predictable). Two of these factors appear to not have been considered in previous works focusing on a large sample of events. Most of these effects are not statistically significant at the 95% level due to the still relatively small sample size, though all would exceed a 90% criteria at least marginally. Combined, however, they account for 40% of the inter‐event spread in SSW predictability, thus indicating that SSWs with favorable precursors are significantly more predictable.

## Key Points

- Predictability of sudden stratospheric warmings (SSWs) is compared among 10 subseasonal to seasonal (S2S) models for 16 events
- Four factors distinguish SSWs with above average predictability: (a) enhanced West Pacific convection due to the Madden‐Julian Oscillation
- (b) Quasi‐Biennial Oscillation easterlies near 50 hPa; (c) displacement SSWs; and (d) stratospheric preconditioning before the event

## 1 Introduction

During a major sudden stratospheric warming (SSW), stratospheric westerly winds in the circumpolar region reverse to easterly winds and temperatures rise over the pole by tens of degrees (Baldwin et al., 2021; Butler et al., 2015; Charlton & Polvani, 2007; Schoeberl, 1978). SSWs are typically followed by anomalous cold air outbreaks in northern Eurasia and enhanced precipitation over Southern Europe and parts of East Asia (Garfinkel et al., 2017; Karpechko et al., 2018; Kolstad et al., 2010; Kretschmer et al., 2018; Lehtonen & Karpechko, 2016; Thompson et al., 2002). As the characteristic time scale of a major SSW and its surface impact extends for several months, accurately predicting SSWs would open a window of opportunity for more reliable probabilistic predictability of surface weather anomalies on subseasonal time scales (Baldwin et al., 2003; Sigmond et al., 2013; Tripathi et al., 2015).

The factors governing the predictability of SSW events are only partially known. Previous work has found that predictability can range from several days to near a month depending on the model used and the specific SSW focused on (Karpechko, 2018; Noguchi et al., 2016; Rao et al., 2019, 2020; Taguchi, 2014; Tripathi et al., 2016). This wide spread may reflect differences in the predictability of different events, in the skill of different forecast systems, and on the method used to quantify successful prediction. For example, raising the model‐lid has been shown to lead to an improved predictability of SSWs (Marshall & Scaife, 2010), and the high‐top subseasonal to seasonal (S2S) models examined by Rao et al. (2019), Domeisen et al. (2020), and Rao et al. (2020) typically performed better at capturing SSWs than the low‐top models. Some studies have suggested that split SSWs are more difficult to forecast than displacement SSWs (Domeisen et al., 2020; Taguchi, 2016a, 2018, 2020), though because of the limited sample size the statistical significance of this effect is relatively weak. Accurately capturing the anomalous wave flux in both the troposphere and lower stratosphere that usually precedes SSWs has also been pinpointed as important for SSW predictability (Karpechko et al., 2018; Mukougawa et al., 2005; Taguchi, 2016b, 2018; Tripathi et al., 2016). Relatedly, the easterly deceleration leading up to SSWs was more predictable in the two models considered by Garfinkel and Schwartz (2017) for SSWs preceded by the phase of the Madden Julian Oscillation (MJO) with enhanced convection in the western Pacific, the phase that has been shown to lead to more SSWs overall (Garfinkel et al., 2012).

Additional factors have been noted to help induce a SSW, though their role for predictability is still not clear. For example, a stronger waveguide for Rossby waves due to a poleward shifted and accelerated vortex accompanied by weaker westerlies in the subtropics leads to a better defined surf‐zone and precedes many SSWs (Baldwin & Holton, 1988; Lawrence & Manney, 2020), though whether such SSWs are more predictable has not been explored in a large sample of SSWs. In addition, the easterly phase of the Quasi‐Biennial Oscillation (QBO) leads to more SSWs, and while case studies have suggested that SSWs during the easterly phase of the QBO may be more predictable (Rao et al., 2019, 2020, 2021), this relationship has not been explored in a large sample of SSWs.

The S2S Prediction project (Vitart et al., 2017) has recently made available a large number of hindcasts and accompanying operational forecasts covering the past few decades. These simulations are all initialized with observed sea surface temperatures and the atmospheric state, and as they are used operationally, they can be compared directly to observed variability during the duration of their forecast. There are two previous studies which contrasted multi‐model predictability of different specific SSWs in the S2S database. Taguchi (2018) considered 4 NH SSWs in the hindcasts of 9 models, while Taguchi (2020) considered 10–11 NH SSWs in the hindcasts of 4 models, and noticed that the predictability of the SSW varies with event types (vortex split or displacement), the model considered, and the ability to represent the anomalous heat flux. Here we revisit the S2S database and considering ten models and 16 different major SSWs, a larger sample than any previous study, we attempt to answer the following question: what distinguishes SSWs that were well‐forecasted from those that were poorly forecasted?

We demonstrate that regardless of the metric used, predictability for SSWs varies from less than five days to almost 20 days depending on the SSW in question. This spread in predictability is associated with a range of factors, including two which appear to have been seldom demonstrated before in such a large sample of events: preconditioning of the vortex and lower stratospheric easterly QBO conditions.

## 2 Data and Methods

We focus on the 10 modeling centers that have contributed to the S2S Prediction project (Vitart et al., 2017) with output at 10 hPa—the Australian Bureau of Meteorology (BoM), the European Centre for Medium‐Range Weather Forecasts (ECMWF), the China Meteorological Administration (CMA), the United Kingdom Met. Office (UKMO), the National Center for Environmental Prediction (NCEP), the Korean Meteorological Agency (KMA), the Japan Meteorological Agency (JMA), the Institute of Atmospheric Sciences and Climate of the National Research Council of Italy (ISAC‐CNR), Environment and Climate Change Canada (ECCC), and Meteo France (CNRM). Table 1 summarizes the models analyzed in this study. We use the high‐top version of CMA starting in 2004 when its hindcasts are first available, and the low‐top version earlier when the high‐top version is unavailable. These various models differ in the quality of their representation of the stratosphere: the stratosphere is less well resolved in BoM and ISAC‐CNR as compared to the other models (Table 1). Note that we use the high‐top version of ECCC, and download the once‐weekly hindcasts issued both in 2020 and in 2021 to increase temporal resolution to twice‐weekly. For the UKMO, we downloaded hindcasts for the operational model in use during 2015 and the winter of 2019/2020, and for the ECMWF, we downloaded data for the model version in use during 2016 and the winter of 2019/2020 (CY41R1/CY41R2 and CY46R1). Real‐time forecasts are used for the three SSWs since 2018.

**Table 1:** For the UKMO, We Downloaded Hindcasts for the Operational Model in Use During 2015 and the Winter of 2019/2020, and for the European Centre for Medium‐Range Weather Forecasts, We Downloaded Data for the Model Version in Use During 2016 and the Winter of 2019/2020 (CY41R1/CY41R2 and CY46R1)

S2S model experiments chosen model (ensemble members)	vertical levels	model top
CMA: BCC‐CPS‐S2Sv1 (4)	40	0.5 hPa
CMA: BCC‐CPS‐S2Sv2 (4)	56	0.1 hPa
NCEP (4)	64	0.02 hPa
ECMWF2016 (11)	91	0.01 hPa
ECMWF2019 (11)	91	0.01 hPa
BoM (33)	17	10 hPa
UKMO2015 (3)	85	85 km
UKMO2019 (7)	85	85 km
KMA (3)	85	85 km
M$\overset{´}{e}$t$\overset{´}{e}$o France: CNRM‐CM 6.1 (10)	91	0.01 hPa
CNR‐ISAC (5)	54	6.8 hPa
ECCC: GEPS6 (4)	45	0.1 hPa
JMA: GEPS1701 (5)	128	0.01 hPa

*Note.* These versions are considered separately. Note that the low top CMA version is used for 1998 through 2003, and the high‐top CMA version since 2004.

**Figure 1:**
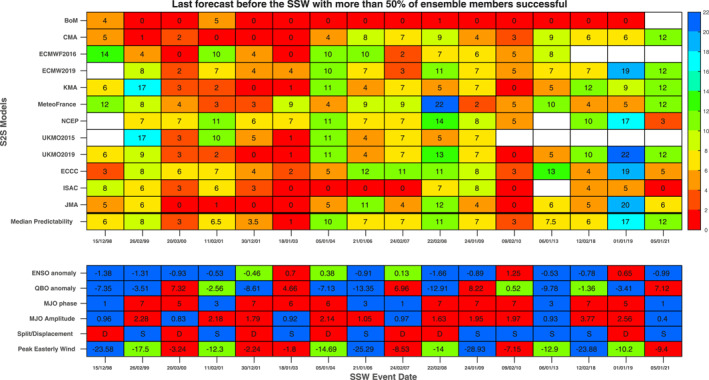
Summary of predictability for all 16 Sudden stratospheric warmings (SSWs) and 10 models considered in this work. The largest number of days before the SSW in which at least half of the hindcast ensemble members still simulate a SSW (working backwards from the actual SSW date) is indicated. A “0” indicates that this criterion is not met by any initialization prior to the SSW onset date. Institute of Atmospheric Sciences and Climate (ISAC) and National Center for Environmental Prediction (NCEP) hindcasts are not archived on the subseasonal to seasonal servers for the 2013 event. Note that the low‐top version of China Meteorological Administration (CMA) is used for SSWs in 2003 or earlier, as the hindcasts for the high‐top CMA begin only in 2004. The median predictability excludes Bureau of Meteorology, and also CMA before 2004, as these models are known to suffer from large mean state biases (Lawrence et al., 2022; Schwartz & Garfinkel, 2020; Schwartz et al., 2022). Also indicated are the El Nino‐Southern Oscillation (ENSO), Quasi‐Biennial Oscillation (QBO), and Madden Julian Oscillation (MJO) conditions preceding the SSW, as well as the SSW morphology and peak easterly winds in the 2 weeks after the onset. For ENSO and the QBO, these are color‐coded based on their sign (with neutral in green). For the MJO, Phase 5/6/7 and amplitudes exceeding 1 are colored red. Displacement events are also colored red. Peak easterly winds between 0 and −10 m/s or less than −20 m/s are colored red and blue respectively, with intermediate events in green.

We focus on 16 SSWs that have occurred since 1998, ten of which occurred in the period common to all models (1999–2009). These SSWs are listed in Figure 1. For each event, we also consider the El Nino‐Southern Oscillation (ENSO), MJO, and QBO phase immediately before the event. The ENSO state is characterized using the observed Niño3.4 index extracted from monthly mean ERSSTv5 data (Huang et al., 2017) for the calendar month which contains the onset date of the SSW. The MJO state is defined following Wheeler and Hendon (2004), and specifically we compute the average amplitude and phase using the two Real‐time Multivariate MJO Indices from 5 to 15 days before the SSW in order to characterize the MJO state preceding a SSW (motivated by Garfinkel et al., 2012). If the amplitude is below 1.0, then the MJO is considered to be inactive. The QBO state is characterized using the observed zonal mean zonal wind at 50 hPa in monthly mean NCEP CDAS reanalysis data for the calendar month which contains the day of the SSW. The characterization of a SSW as either split or displacement, and also the onset date of the event, follows Table 1 of Cohen and Jones (2011) for earlier events, Tripathi et al. (2016) for the 2013 event, and Rao et al. (2020) and Rao et al. (2021) for the three most recent events. All of these aforementioned factors that may potentially lead to enhanced predictability are poorly correlated with each other for the 16 SSW events considered here (the highest correlation in absolute value is −0.32 between the vortex precursor and the QBO), and hence these factors are all treated as independent external drivers that can lead to vortex predictability. The maximum correlation is even lower if we remove the two most marginal SSW events on 30 December 2001 and 18 January 2003 in which winds barely reversed to easterlies (Figure 1). (The suddenness metric introduced later is well correlated with the QBO, however the suddenness metric is not well correlated with predictability as defined using the hit rate metric.) Note that the first day of easterly winds can differ among reanalysis products, however for these events modern reanalyses agree to within 1 day of each other (e.g., Butler et al., 2017).

An ensemble member is deemed “successful” if it simulates a SSW within ±3 days of its actual onset date. This definition of a “success” follows Taguchi (2016a), Taguchi (2020), Domeisen et al. (2020), Rao et al. (2019), and Rao et al. (2020). In addition to this hit rate metric, we consider the absolute error, that is, the absolute value of the difference between the ensemble mean predicted zonal wind at 60°N, 10 hPa and the actual zonal wind, averaged within ±1 day of the observed onset date. A “successful” forecast requires the absolute error to be less than 10 m/s, and results are similar if we use a threshold of 5 m/s instead (not shown).

## 3 Results

**Figure 2:**
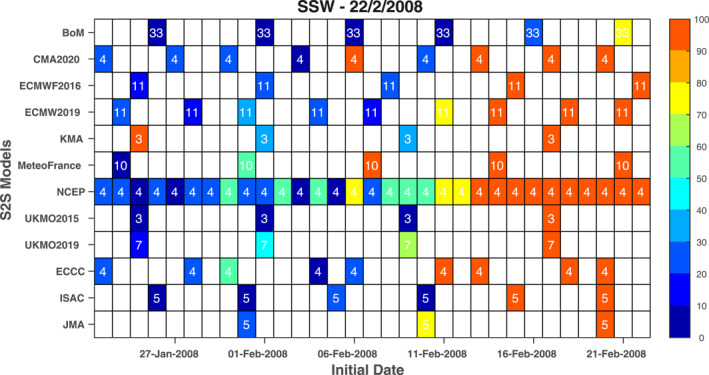
Initialization dates and ensemble sizes of the hindcasts available from each subseasonal to seasonal model from 22 January to 22 February 2008. The ensemble size is indicated by the number in each grid cell. The color shading in each grid cell denotes the sudden stratospheric warming hit ratio (units: %) of the ensemble members that forecast a reversal of the zonal mean zonal wind at 60°N and 10 hPa from 19 to 25 February 2008 (i.e., a maximum error of ±3 days is allowed). A blank grid denotes that no hindcasts were initialized on the specific day for the corresponding model.

We begin with a map of the hit‐rate for the SSW that occurred on 22 February 2008 in Figure 2, as this SSW turned out to be the most predictable one of the SSWs considered in this study (as defined by median hit rate among the models) not previously documented, and the only SSW with predictability approaching that of the well‐predicted 1 January 2019 event already documented by Rao et al. (2019). Nearly all models successfully simulated this SSW for initializations up to 10 days before the SSW. For several models hit rates exceeding 50% are present 15 days before the SSW. All MeteoFrance hindcasts initialized 15 days before the SSW capture it, while half of the MeteoFrance hindcasts initialized 22 days before the SSW still successfully simulate it. The net effect is that the predictability of this event (as for the 1 January 2019 event) for some modeling systems substantially exceeds the deterministic predictability limit of around 10 days for SSWs commonly mentioned in previous work (Domeisen et al., 2020; Taguchi, 2020). In contrast, other SSWs, for example, the 18 January 2003 event, are poorly predicted (Figure S1 in Supporting Information S1).

Similar maps of the hit‐rate have been created for all 16 SSWs, and Figure 1 summarizes the predictability of each SSW for each forecast system. Specifically, we list the earliest forecast lead day on which at least 50% of the ensemble members still successfully forecast the SSW. The frequency with which hindcasts are produced and the specific hindcast dates differ among the models, and hence it can be challenging to directly compare forecast skill between models with, say, daily hindcasts to models with hindcasts every 10 days. Nevertheless, there is a general indication that the low‐top models (BoM and ISAC‐CNR) struggle as compared to the high‐top models, in agreement with previous work. Relatedly, the CMA modeling system is more successful at simulating SSWs starting in 2004 than for earlier SSWs when only the low‐top version of CMA is available. The eight high‐top models differ in their skill for different events, and given the lack of consistent initialization dates, we do not discuss their relative abilities to represent the timing of SSW onset nor grade them. Rather, our goal going forward is to distinguish between SSWs that are relatively more predictable versus relatively less predictable, defined as the median predictability among the models. Results are similar if we focus on whether the “success” rate is greater than the “false alarm rate,” defined as the number of members that predict an event to occur within versus outside of ±3 days surrounding the actual event (not shown). Results are also similar for the absolute error metric (Section 2) if marginal SSWs are not included, as discussed later.

We begin with the three relatively predictable SSWs during the common period to all models (1999–2009): the 22 February 2008, 26 February 1999, and 5 January 2004 events. All three of these events occurred during MJO Phase 6 or 7, and two occurred during strong eQBO conditions and the third during weak eQBO conditions. Both eQBO and the MJO Phase 6 and 7 are known to lead to a weaker vortex, and hence are plausibly linked to enhanced SSW predictability. In contrast, two of these three occurred during La Nina and the third during neutral ENSO, which tends to lead to a stronger monthly mean vortex and a reduced probability of easterly winds in the full hindcast ensemble for the models considered by Garfinkel et al. (2019). However, over the period considered in this study the observed relationship between ENSO and SSW was opposite, with more SSWs during La Nina (Domeisen et al., 2019), and ENSO also may modulate the impact of the MJO on SSWs (Ma et al., 2020). Favorable precursors ‐ in particular El Nino, MJO Phase 5, and eQBO–were present before the well‐predicted 1 January 2019 event as well (Rao et al., 2019).

**Figure 3:**
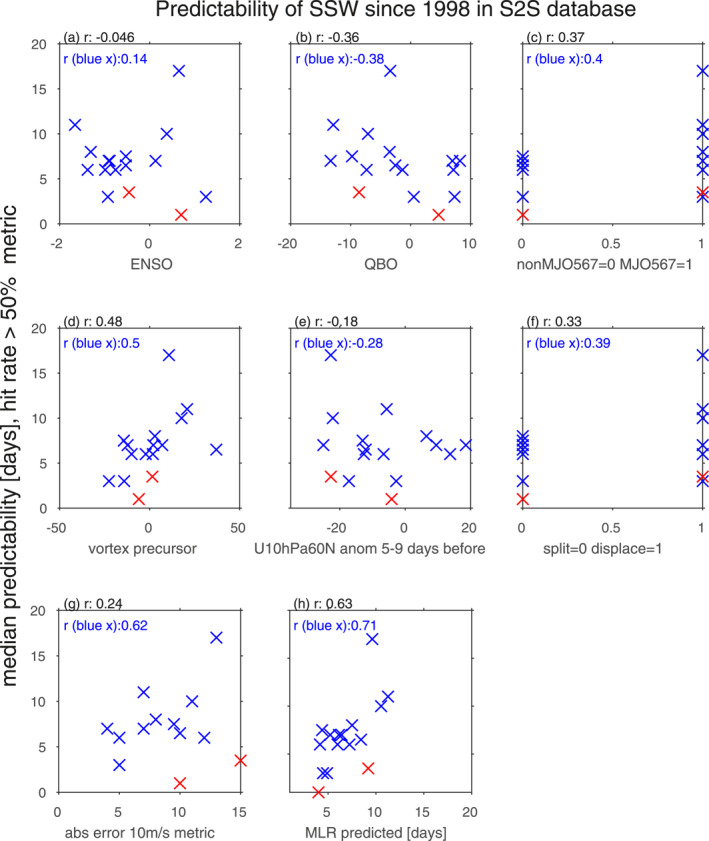
Scatter plots comparing the median predictability as defined using the hit rate exceeding 50% metric (*y*‐axis) to each of the following factors (*x*‐axis): (a) Niño3.4 index [Kelvin]; (b) Quasi‐Biennial Oscillation [m/s]; (c) whether the event was preceded by Madden Julian Oscillation Phase 5, 6, or 7 of amplitude exceeding 1 in the two weeks before the event; (d) precursor pattern in the subpolar upper stratosphere 15–19 days before the sudden stratospheric warming (SSW) using ERA5 reanalysis data [m/s]; (e) U10 hPa, 60N anomalies 5–9 days before the SSW using ERA5 reanalysis data [m/s]; (f) split versus displacement; (g) comparison to absolute error of 10 m/s metric (Figure S2 in Supporting Information S1); (h) a multiple linear regression model using panels b, c, d, and f as given by Equation 1 [days]. Each of the 16 SSWs is indicated with an “x”, and the two weakest, most marginal SSWs are indicated in red (18 January 2003 and 30 December 2001). The correlation for each panel is indicated both with and without including these two marginal SSWs. For the 2021 SSW, the median predictability does not include predictions from European Centre for Medium‐Range Weather Forecasts and MeteoFrance, as these modeling centers significantly upgraded their model as compared to the model versions used for all other SSWs.

These results are generalized to all SSWs in the scatter plots on Figure 3. These compare the median predictability for each event to these long‐duration external forcings. While ENSO is not related to the predictability of SSWs, the QBO and MJO state are (Figures 3a–3c): eQBO and the occurrence of MJO phases 5, 6, 7 of amplitude exceeding 1 are associated with more predictable SSWs, though the correlations do not meet the threshold for statistical significance using a two‐tailed students‐t test for 14 degrees of freedom (namely, *r*(*α* = 0.05) = 0.5). Even if we were to use a one‐tailed test motivated by previous work that has demonstrated that these modes of variability lead to a weaker vortex, the threshold for significance would be 0.43 and we still could not reject a null hypothesis of no effect; a Monte‐Carlo bootstrapping in which (as an example) the QBO values are randomly re‐assigned to a different SSW 20,000 times indicates an essentially identical threshold (Figure S4 in Supporting Information S1). A Wilcoxon rank sum test of the effect of MJO Phase 5/6/7 on hit rate also is not high enough to reject the null hypothesis of no effect at the 95% level. However both of these correlations would be sufficient to reject the null hypothesis if a confidence level of 90% was used. The importance of the MJO and eQBO is somewhat weaker for the absolute error metric (Figure S3 in Supporting Information S1). While the effect of the MJO on SSW predictability for the metrics used in this study is, at best, marginally significant, other metrics do show a significant impact of the MJO on SSW predictability: Garfinkel and Schwartz (2017) found that models predicted a significantly stronger deceleration of winds for SSWs preceded by MJO Phase 6/7 as opposed to other events, though the deceleration they found does not necessarily lead to a wind‐reversal as observed.

Thus far we have found some evidence that long‐duration external forcings can contribute to SSW predictability, and now we switch our focus to whether the properties of the SSW itself help distinguish well‐predicted SSWs from poorly predicted SSWs. Specifically, some are preceded by stratospheric preconditioning while others are not (Lawrence & Manney, 2020). For some the weakening of the westerlies is more gradual while others are more sudden, and finally some SSWs are splits while others are displacement. Is one type of event harder to predict?

**Figure 4:**
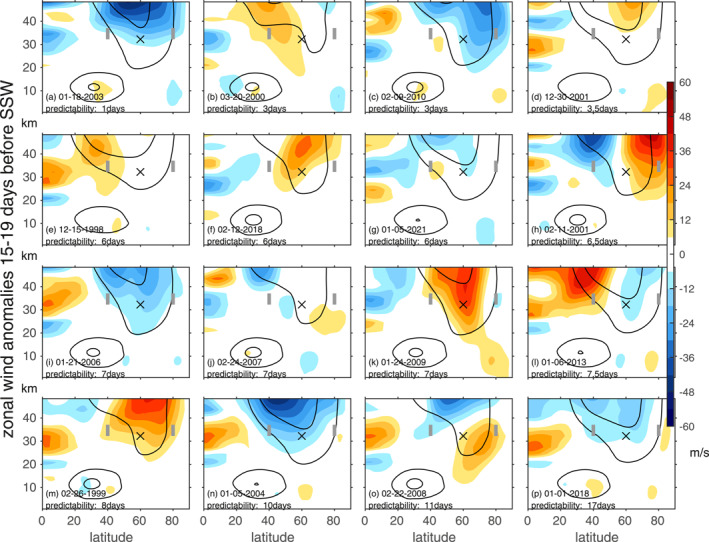
Latitude‐height cross sections of zonal wind anomalies 15–19 days before the 16 sudden stratospheric warmings in this paper using ERA5 reanalysis data, ordered by their respective median predictability as given by the hit‐rate metric of Figure 1. An “x” marks the location of 10 hPa, 60°N, and vertical gray lines mark the locations used to define stratospheric preconditioning. Black contours denote the 20 m/s and 40 m/s climatological isotachs for the corresponding days.

We begin by considering the state of zonally averaged zonal wind in days 15–19 before each of these SSW events using ERA5 reanalysis data in Figure 4, with the SSWs ordered by the median hit rate predictability metric. First, note that the five most predictable SSWs (panels l through p) all occurred during eQBO conditions in the tropical lower stratosphere (though as shown in Figure 3b so did several poorly predicted SSWs). Previous work has shown that SSWs are often preceded by a poleward shift of the stratospheric vortex and a weakening of winds in the subtropics, which has the effect of bringing the waveguide for Rossby waves closer to the pole while also strengthening it. We diagnose this effect by computing the difference between zonal wind at 80°N, 5–10 hPa and zonal wind at 40°N, 5–10 hPa (indicated with gray vertical lines in Figure 4). This vortex preconditioning metric is compared to the median predictability metric in Figure 3d, and it is clear that there is a strong connection that just misses the 95% threshold for significance for a two‐tailed test (either using a Monte‐Carlo bootstrapping or a Student‐t test; Figure S4 in Supporting Information S1). However, this connection would be deemed significant if a one‐tailed test was used, and such a one‐tailed test may be appropriate here due to a priori knowledge that vortex preconditioning should help SSW development. Results are similar for days 10–14, or for the absolute error of 10 m/s metric, though below the threshold for significance.

One might hypothesize that SSWs which develop more slowly (i.e., easterly U10 hPa 60°N anomalies are present well before the onset date) should be easier to simulate, as the initialization already includes a vortex weakening. We examine this hypothesis in Figure 3e, which compares 10 hPa, 60°N zonal wind anomalies 5–9 days before onset using ERA5 reanalysis data (i.e., a “suddenness” metric) to the median predictability. While there is a tendency for SSWs that already feature weakening of the vortex 5–9 days before onset to have a higher hit rate than SSWs that develop more suddenly, the overall correlation is not significant. The importance of slowly developing easterlies is more important for the absolute error of 10 m/s metric (correlation of −0.36, Figure S3 in Supporting Information S1), which is high enough to confidently reject a null hypothesis of no effect at the 90% level. Note that this metric is well correlated with the QBO across these 16 events (correlation of 0.54, whereby wQBO SSWs tend to be preceded by strong vortex states), however, hence there is some ambiguity as to whether the QBO or the suddenness metric are more important for SSW predictability when using the absolute error metric.

Finally, we consider vortex morphology. Figure 3f shows that displacement SSWs are more predictable than split SSWs as measured by the hit rate. If we focus on the absolute error, the correlation increases to 0.46 and the Wilcoxon rank sum test is now at the threshold for significance at the 95% level if a one‐tailed test is used.

The predictability of SSWs as derived from the hit‐rate and absolute error metrics are compared in Figure 3g. The relatively low correlation (0.24) is heavily influenced by two outlier SSWs (marked in red–30 December 2001 and 18 January 2003), and if these two events were removed then the correlation between the metrics rises to 0.62. Both have lower hit‐rates than would be expected given their absolute errors. (Note that results earlier in this paper concerning factors leading to enhanced predictability are robust to removing these two events.) Why might these events have a poor hit‐rate despite a relatively successful absolute error? These two events were the weakest SSWs of the 16 considered, and in order to clarify why this matters, we focus specifically on the 30 December 2001 event, the weaker of the two. Note that this event was poorly predicted using the hit‐rate metric despite many favorable precursors (e.g., a strong MJO Phase 6/7 event) but was the most predictable SSW if the absolute error metric is used (Figure S2 in Supporting Information S1), and indeed was used by Garfinkel and Schwartz (2017) as a case‐study of how ensemble members which successfully simulate MJO‐related convection tend to better predict the ensuing SSW.

**Figure 5:**
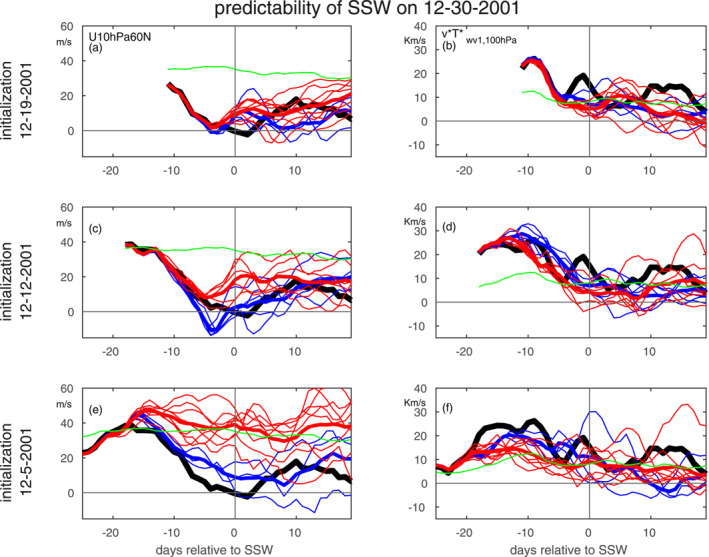
Evolution of the 12‐30‐2001 sudden stratospheric warming (SSW) in the European Centre for Medium‐Range Weather Forecasts forecast system. ERA‐I reanalysis data are shown in thick black, relatively successful ensemble members in blue (defined as those with a wind reversal on the SSW onset date for the top two rows, and those in which U10 hPa 60°N was below 20 m/s on the SSW onset date for the bottom row), poorer ensemble members in red, and the daily climatology in reanalysis in green. Thick blue and red lines show the ensemble means for the more‐successful and less‐successful ensemble members respectively. (left) zonal mean zonal wind at 10 hPa, 60N; (right) wave‐1 heat flux at 100 hPa from 40 to 80°N. We show the wave‐1 heat flux only as this was a displacement event.

We focus on ECMWF hindcasts of this event in Figure 5, with relatively successful ensemble members in blue and other ensemble members in red. On 26 December 2001, zonal winds at 10 hPa, 60N in ERA‐I weakened to 2.2 m/s, though only four days later did they actually reverse. Three of the eleven ECMWF initializations from 19 December 2001 simulated a SSW on 26 December 2001 (indicated in blue), and the ensemble mean vortex strength was weaker than observed (Figure 5a). If we focus on the 12 December 2001 initialization, five of the eleven ensemble members simulated a SSW within three days of 26 December 2001 (indicated in blue), and again the ensemble mean vortex strength was more easterly than observed (Figure 5c). Only the 5 December 2001 initialization can be considered an unambiguous forecast bust: most ensemble members struggle to simulate a weakening of the vortex, and only one member simulates a SSW (Figure 5e). While the 19 December 2001 and 12 December 2001 initializations capture the extremely strong pulse of heat flux in the first week (and over‐estimate it for the successful ensemble members initialized on 12 December 2001), the pulse immediately before the SSW is not well represented even in the relatively successful ensemble members, and this late developing pulse appears to have been important for the winds reversing on 30 December 2001. The mid‐December pulse of heat flux is under‐estimated by the 5 December 2001 initialization, though the three ensemble members that more realistically simulate a weakening of the vortex also do a better job at capturing this wave flux event. The net effect is that the 30 December 2001 SSW was preceded by a strong and long‐lasting wave pulse which is generally well represented even in initializations 18 days before the event, however the eventual SSW was comparatively weak and the models struggled to capture its timing due to their failure to capture the late December secondary heat flux pulse. This struggle to capture its timing led to a forecast bust if we adopt the ±3 days criteria used by previous work (Domeisen et al., 2020; Taguchi, 2016a, 2020), however the absolute error metric reveals this as the most predictable SSW. Such events are the exception, however, and this divergence between the metrics is associated with the relative weakness of the underlying SSW; for non‐marginal SSWs the absolute error and hit‐rate metrics generally agree.

Finally, we consider the question: if all of the favorable precursors are considered together, can they successfully predict which SSW events are more predictable? To answer this question, we form a multiple linear regression model using these four predictors: the QBO, MJO, morphology, and vortex precursor (panels b, c, d, and f of Figure 3) to predict the hit ratio metric. Specifically we solve for the *β* coefficients in the equation

(1) $${\text{hit}\;\text{rate}}_{\text{predicted}}\left(m\right)=\beta_{0}+\beta_{\text{QBO}}\cdot \text{QBO}\left(m\right)+\beta_{\text{MJO}}\cdot \text{MJO}\left(m\right)+\beta_{\text{morphology}}\cdot \text{morph}\left(m\right)+\beta_{\text{vortex}}\cdot V\left(m\right).$$

that minimize the residual of the resulting best fit line using ordinary linear least squares regression across all 16 SSW events, with *m* ranging from 1 to 16. All four predictors are standardized so that the units of all four *β* are identical.

Figure 3h compares the actual predictability from the models to the predicted hit ratio from the multiple linear regression model. It is clear that the multiple linear regression model can explain much of the inter‐event variability in predictability (correlation of 0.63, significant at above the 99% level using either a one‐tailed Student‐t or a bootstrapping test; Figure S4 in Supporting Information S1). The coefficients from the multiple linear regression model indicates the relative importance of each of the four. The most important is the vortex precursor, with a 1.4 days increase in predictability for every 1 standard deviation increase in the vortex precursor metric (i.e., *β*_vortex_ = 1.4 days per std). The next most important is the MJO, with a 1.1 day increase in predictability for every 1 standard deviation change in the MJO metric (i.e., *β*_MJO_ = 1.1 days per std). The last two coefficients, *β*_QBO_ and *β*_morphology_, are −0.62 and 0.75, respectively. The relative importance of these predictors differs if one focuses on the absolute error metric (the morphology predictor is now most important with *β*_morphology_ = 1.29 days per std), and further the correlation of the predicted absolute error with the actual absolute error using these four predictors drops to 0.53. However replacing the QBO predictor with the suddenness predictor leads to a improved correlation of 0.58 (Figure S3 in Supporting Information S1). Regardless of the metric used to quantify predictability, the net effect is that SSW events preceded by favorable precursors are more predictable.

## 4 Summary and Discussion

SSWs are associated with a range of surface impacts, and to the extent that SSWs can be predicted on subseasonal timescales, there is hope that the subsequent surface impacts could be predicted at earlier leads. Here, we evaluated the predictability of a larger sample of SSWs than has been considered in any previous work, and showed that there is wide diversity in the predictability of different SSW events. Some are predictable 20 or more days in advance in the best performing models, while others can only be predicted a week in advance even in the best performing models. Previous work using fewer models and fewer cases has focused on the vortex morphology or the existence of tropospheric precursors as important for SSW predictability, with displacement events and events preceded by the MJO Phase 6/7 more predictable (Domeisen et al., 2020; Garfinkel & Schwartz, 2017; Rao et al., 2019; Taguchi, 2018). Our results with a relatively larger number of models and SSWs (10 models and 16 SSWs) support this previous work: SSWs preceded by MJO Phase 6/7 and that are of displacement morphology are indeed more predictable. In addition to these factors, our results also provide evidence for two factors that do not seem to have been noted before. Specifically, SSWs preceded by a tightening of the vortex around the pole and a retraction from the subtropics, and also SSWs during eQBO, are more predictable. There is also an indication that more gradual SSWs are more predictable especially if an absolute error metric is used. Despite the larger sample assembled here than in previous work, these effects are capable of clearly rejecting a null hypothesis of no effect only at the 90% confidence level for the metrics considered here. Future work should also consider strong deceleration events (e.g., Wu et al., 2022) that nonetheless do not meet the SSW definition in order to enlarge the sample size.

A commonly used criteria (and the criteria used mostly in this work) for a successful forecast is that the central date of the simulated SSW falls within ±3 days of the actual event. While this criteria is simple to apply and logical for strong SSWs, it may lead to an underestimate of skill for more marginal events. For example, ECMWF initializations 18 days before the 30 December 2001 event performed remarkably well for the first 2 weeks of the forecast (Figure 5) with low absolute errors (Figure S2 in Supporting Information S1), yet the ±3 days hit‐rate criteria judges this forecast to be a failure. This was a marginal SSW event, however, and if this event (and also the 18 January 2003 marginal event) are not considered, the hit rate metric and the absolute error metric agree on the relative predictability of most other events.

An important caveat of our study is that we are assuming that the currently realized predictability of SSWs from biased forecast systems (Lawrence et al., 2022; Schwartz & Garfinkel, 2020; Schwartz et al., 2022) is a proxy for the “true” potential predictability of these SSW events. While our computation of median predictability only considers high‐top models which are known to be less biased (Lawrence et al., 2022; Schwartz & Garfinkel, 2020; Schwartz et al., 2022), future work should reconsider future generations of high‐top models with smaller biases. This assumption is particularly suspect for the marginal SSW events, as for example, a relatively small too‐strong vortex bias can lead to the forecast system missing the transition to easterlies (Figure 5). While the vortex is well‐represented in the ECMWF forecast system (Figure 7 of Schwartz et al. (2022)), biases exist for other systems and future work should explore the impact of bias correction on prediction skill of different SSWs.

Regardless of whether we include or exclude marginal SSWs from the sample, we find evidence that there are four distinct factors, of roughly equal importance and not well‐correlated with each other, that help lead to more predictable SSWs: MJO Phase 5/6/7, easterly QBO, displacement morphology, and a strong mid‐ and upper‐stratospheric preconditioning. While individually they are only marginally significant in the still‐small sample available, taken together they are robustly associated with more predictable SSWs, and specifically explain more than 40% of the inter‐event variability in predictability. Future work should consider whether this enhanced predictability can help lead to improved surface forecasts.

## Supporting information

Supporting Information S1

## Data Availability

The original S2S database is hosted at ECMWF as an extension of the TIGGE database, and can be downloaded from the ECMWF server verthttp://apps.ecmwf.int/datasets/data/s2s/levtype=sfc/type=cf/vert. The QBO data was downloaded from the NCEP website verthttps://www.cpc.ncep.noaa.gov/data/indices/qbo.u50.indexvert. The real time multivariate index of Wheeler and Hendon (2004) was downloaded from the BoM website (http://www.bom.gov.au/climate/mjo/graphics/rmm.74toRealtime.txtvert).
