# Supplementary material for: Which Sudden Stratospheric Warming Events Are Most Predictable?
Source: J Geophys Res Atmos. 2022 Sep 16;127(18):e2022JD037521. doi: 10.1029/2022JD037521 (PMC9540765; doi:10.1029/2022JD037521)
Supplement: Supplementary file 1 — Supporting Information S1 [file JGRD-127-e2022JD037521-s001.pdf]

JOURNAL OF GEOPHYSICAL RESEARCH

Supporting Information for "Which Stratospheric Sudden Warming Events are Most Predictable?"

Dvir Chwat[1], Chaim I. Garfinkel[1], Wen Chen[2,3], Jian Rao[4]

[1]Fredy and Nadine Herrmann Institute of Earth Sciences, Hebrew University, Jerusalem, Israel

[2]College of Earth and Planetary Sciences, University of Chinese Academy of Sciences, Beijing, China

[3]Institute of Atmospheric Physics, Chinese Academy of Sciences, Beijing, China

[4]Key Laboratory of Meteorological Disaster, Ministry of Education (KLME), Joint International Research Laboratory of Climate and Environment Change (ILCEC), Collaborative Innovation Center on Forecast and Evaluation of Meteorological Disasters (CIC-FEMD), Nanjing University of Information Science and Technology, Nanjing 210044, China

Contents of this file

1. Figures S1 to S5

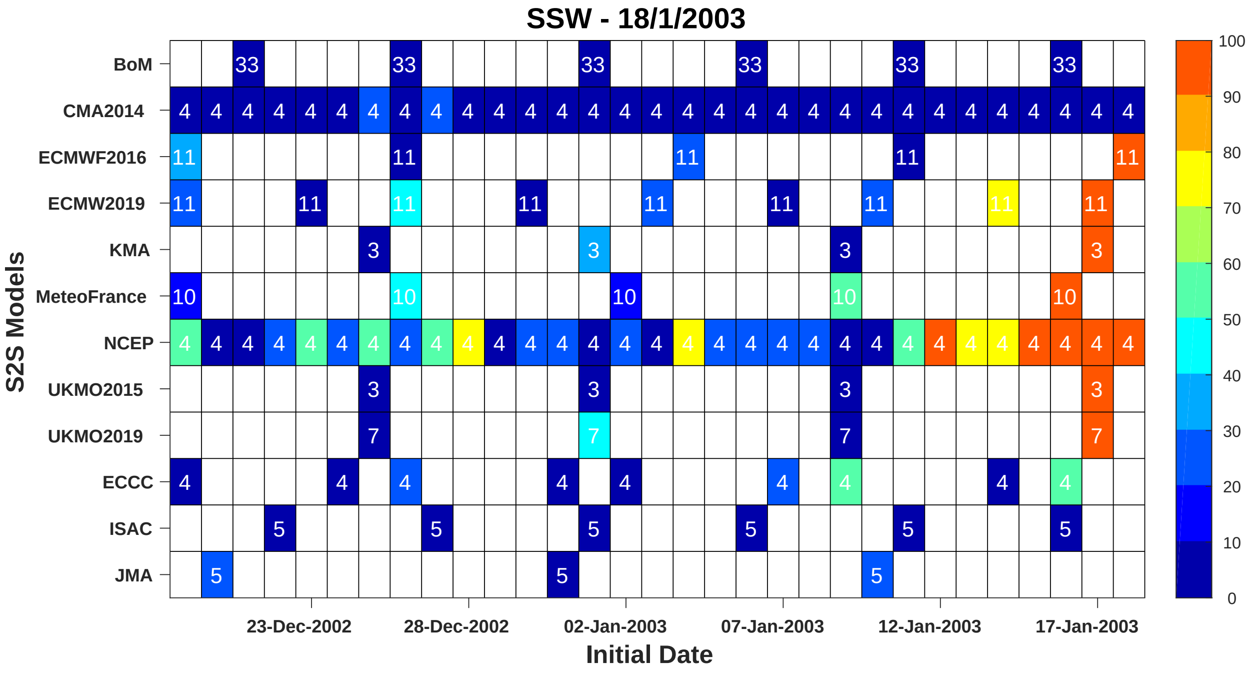

Figure S1. As in Figure 2 of main text but for the January 18, 2003 event.

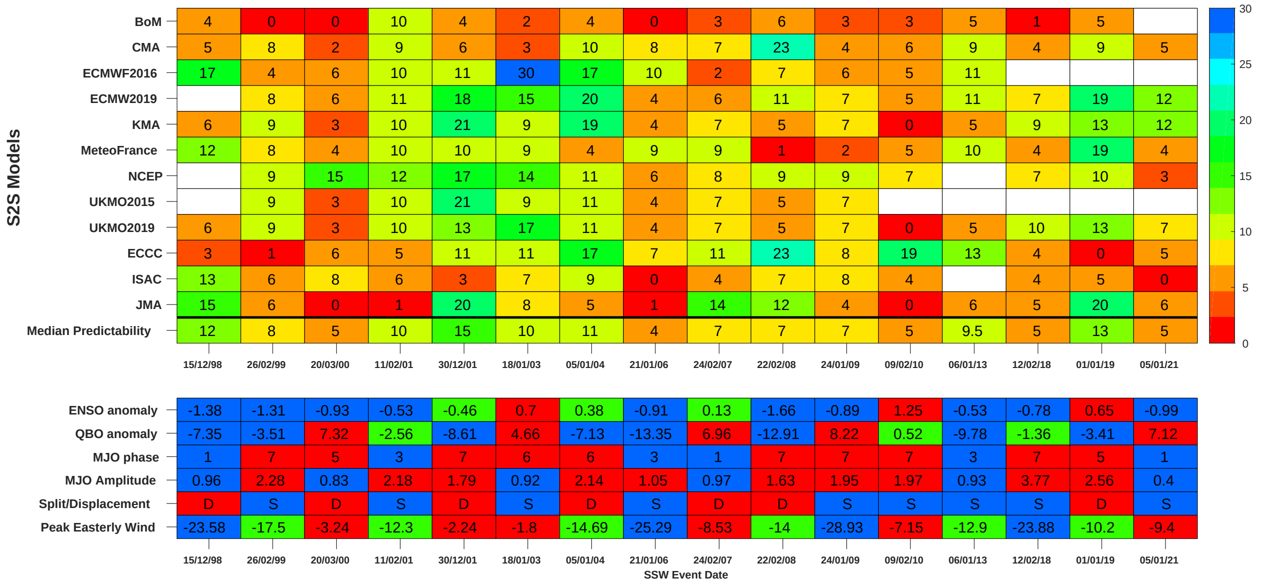

Figure S2. As in Figure 1 of main text but for an alternate method of assessing SSW predictability: the number of days before the SSW in which the absolute error of the ensemble mean U10hPa60N on the onset date is still less than 10m/s (working backwards from the actual SSW date) is indicated.

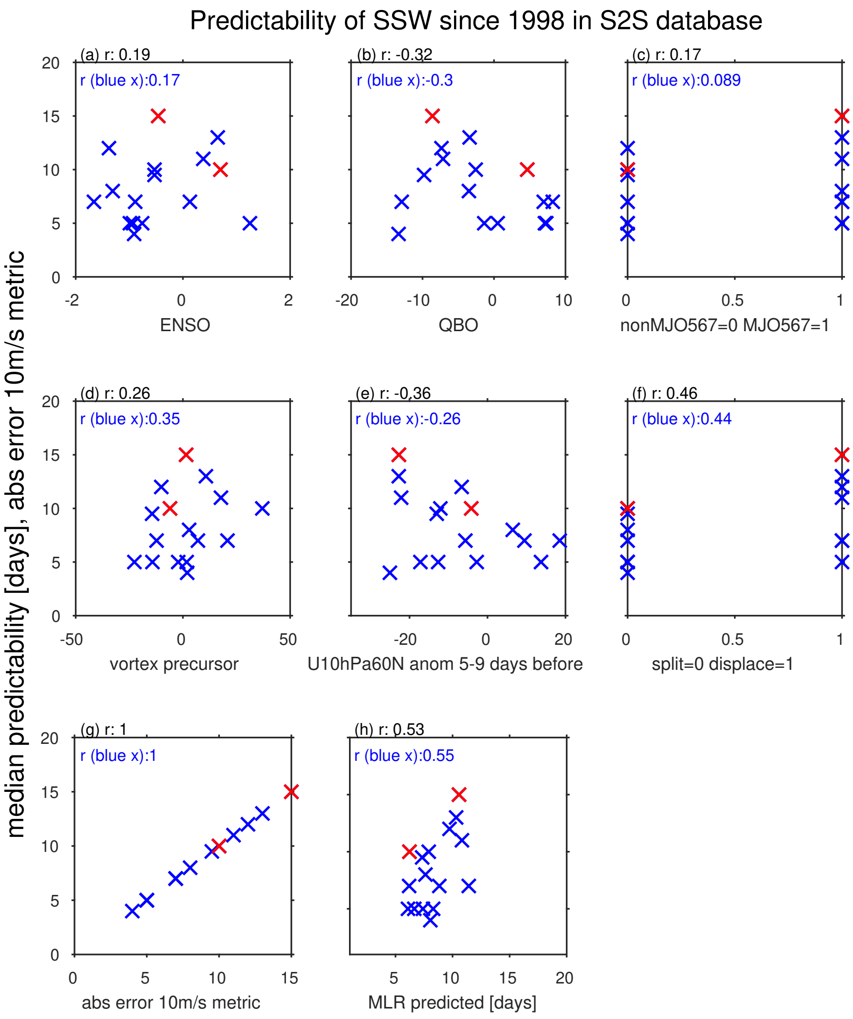

Figure S3. As in Figure 3 of main text but defining median predictability based on the absolute error metric of Figure S2.

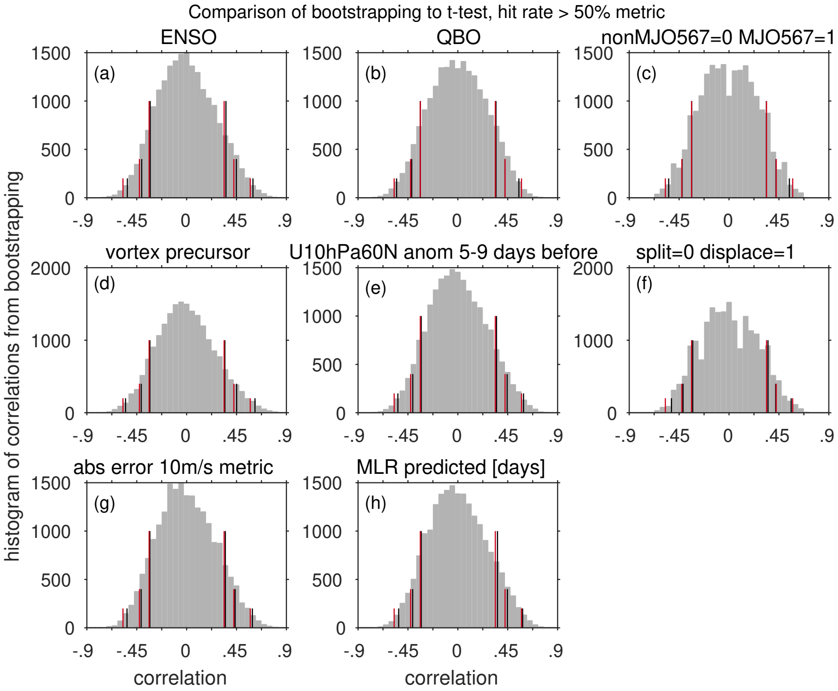

Figure S4. Histograms of the correlations computed from the 20,000 bootstrapping simulations in which (a) ENSO conditions for each of the 16 SSWs are randomly re-assigned. (b) as in (a) but QBO conditions are randomly re-assigned 20,000 times; (c)-(g) as in (a) and (b) but for the quantities shown in Supplemental Figure S3. Vertical black lines indicate the one-tailed 90% threshold (long lines), 95% threshold (medium lines), and 99% threshold (short lines) using the bootstrapping, while vertical red lines indicate the corresponding thresholds using Student-t tests. For most panels the red and black vertical lines overlay and cannot be distinguished.

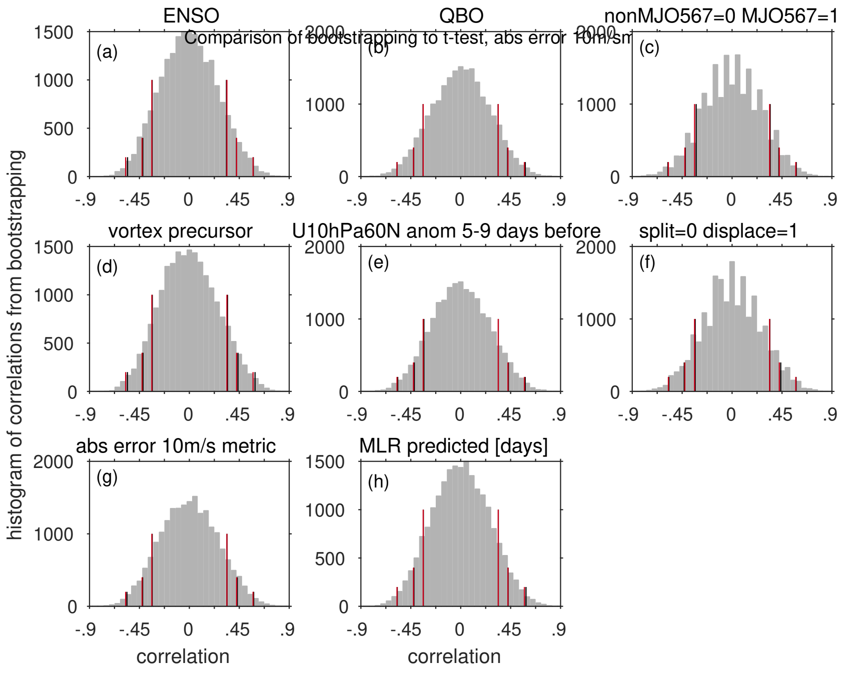

Figure S5. As in Figure S4 but for the absolute error metric.
